# Neurosensory Disturbances and Recovery Patterns in Orthognathic Surgery: A Retrospective Analysis of 579 Cases From a Tertiary Referral Center

**DOI:** 10.7759/cureus.101394

**Published:** 2026-01-12

**Authors:** Masato Narita, Miki Watanabe, Masahiro Okamura, Masashi Iwamoto, Masae Yamamoto, Takeshi Nomura, Akira Katakura, Takashi Kamio

**Affiliations:** 1 Department of Oral and Maxillofacial Surgery, Tokyo Dental College, Ichikawa, JPN; 2 Department of Oral Oncology, Oral and Maxillofacial Surgery, Tokyo Dental College, Ichikawa, JPN; 3 Department of Oral Pathobiological Science and Surgery, Tokyo Dental College, Ichikawa, JPN; 4 Oral and Maxillofacial Radiology, The Nippon Dental University, Tokyo, JPN

**Keywords:** inferior alveolar nerve, neurosensory disturbance, orthognathic surgery, recovery patterns, risk factors, sagittal split osteotomy

## Abstract

Background

Le Fort I osteotomy and sagittal split ramus osteotomy (SSRO) are the most commonly performed procedures for correcting skeletal jaw deformities. Despite improved safety, neurosensory disturbances remain a significant postoperative complication. Comprehensive quantitative assessment of sensory recovery and identification of risk factors are essential for optimizing patient counseling and surgical planning.

Methods

A total of 579 cases with a complete six-month follow-up were analyzed from 642 consecutive orthognathic surgeries performed between 1 April 2020 and 31 March 2025 at the Tokyo Dental College Ichikawa General Hospital. Neurosensory function was assessed using Semmes-Weinstein (SW) monofilament testing at one week and at one, two, three, and six months postoperatively. Normal sensation was defined as the detection of SW 1.65 monofilament, and severe hypoesthesia as failure to detect SW 4.56 or higher. Statistical analyses included chi-square tests, Mann-Whitney U tests, and multivariate logistic regression.

Results

Intraoperative complication rates were 0.4% in the Le Fort I group (n=225) and 2.5% in the SSRO group (n=354). Postoperatively, infraorbital nerve paresthesia occurred in 36/225 cases (16.0%), while inferior alveolar nerve paresthesia occurred in 139/354 left sides (39.3%) and 112/354 right sides (31.6%). Six-month neurosensory recovery rates were 94.4% in the Le Fort I group and 92.9% in the SSRO group, with >85% of recoveries occurring within the first three months. Mandibular advancement of ≥5 mm was the most significant independent risk factor for bilateral inferior alveolar nerve disturbance (left: *p*=0.000292; right: *p*=0.00196). Other complications included maxillary sinus mucosal thickening (14.2%) and metal plate fracture (1.1%).

Conclusion

Orthognathic surgery at this tertiary center demonstrated low intraoperative risk and excellent neurosensory recovery. However, mandibular advancements of ≥5 mm significantly increased the risk of inferior alveolar nerve injury, underscoring the need for enhanced preoperative counseling and surgical planning.

## Introduction

Recent advances in orthognathic surgery have substantially improved procedural safety and reduced complication rates. Nevertheless, intraoperative and postoperative complications remain inherent risks that can adversely affect patient outcomes and quality of life [1-3].

Le Fort I osteotomy and sagittal split ramus osteotomy (SSRO) are the most frequently performed procedures for correcting maxillary and mandibular deformities, respectively. Despite improved safety profiles, complications such as inferior alveolar nerve (IAN) paresthesia, infraorbital nerve disturbance, unfavorable splits, and perioperative adverse events continue to pose clinical challenges [3-5]. Virtual three-dimensional planning and model-based workflows have demonstrably improved surgical accuracy and are increasingly used in orthognathic practice [6].

Previous studies have reported that rates of IAN disturbance after SSRO range from 20% to 85%, with most studies clustering between 30% and 50%. Similarly, infraorbital nerve disturbance after Le Fort I osteotomy is reported in 5% to 16% of cases [7,8]. A clearer understanding of risk factors, recovery trajectories, and preventive strategies is essential to optimize patient care and strengthen preoperative counseling [6-8]. Furthermore, understanding preoperative patient expectations and long-term satisfaction levels is crucial for comprehensive clinical management [9].

This study was conducted at a tertiary referral hospital, which regularly manages complex cases from affiliated institutions. This distinctive patient population offers valuable insights into complication patterns and management strategies in high-risk orthognathic surgery [10].

The present study was performed to assess the incidence, characteristics, and risk factors of intraoperative and postoperative complications in orthognathic surgeries performed at Tokyo Dental College Ichikawa General Hospital over a five-year period, with a particular focus on neurosensory disturbances and their recovery patterns. The primary outcome was the proportion of affected patients that achieved detection of SW 1.65 at six months. Secondary outcomes included time to recovery and identification of independent surgical risk factors. This work was previously presented as a poster presentation at the Japanese Society for Jaw Deformities Annual Meeting in June 2025.

## Materials and methods

Study design

This retrospective cohort study was conducted at a tertiary referral hospital with institutional review board approval (Ethics Review Board approval number I22-14). The study adhered to the principles of the Declaration of Helsinki and relevant local regulations.

Patients

The study population comprised 579 patients with complete follow-up data, selected from 642 consecutive patients who underwent Le Fort I osteotomy or SSRO between April 1, 2020 and March 31, 2025, at the Tokyo Dental College Ichikawa General Hospital, Ichikawa, Japan. The inclusion criteria were an age between 16 and 60 years, availability of complete six-month postoperative follow-up data, and adequate radiographic documentation. The exclusion criteria were a history of facial trauma or prior orthognathic surgery, congenital craniofacial syndromes, incomplete medical records, and pre-existing neurological disorders affecting facial sensation.

Surgical procedures

All operations were performed by experienced maxillofacial surgeons using standardized techniques under general anesthesia with nasotracheal intubation. Le Fort I osteotomies were carried out via intraoral approaches with preservation of the infraorbital nerve [11]. The standard maxillary osteotomy technique involved a horizontal cut 5 mm above the root apices, extending from the piriform rim to the zygomatic buttress bilaterally, with pterygomaxillary separation achieved using a curved osteotome. The maxilla was repositioned according to preoperative surgical planning and stabilized with titanium miniplates and screws. SSRO was performed in accordance with the Obwegeser-Dal Pont technique with modifications as clinically indicated, utilizing ramus osteotomy [12,13]. The proximal and distal segments were repositioned and fixated with titanium miniplates and screws.

Data collection

Data were retrospectively extracted from hospital medical records, outpatient charts, operative reports, and radiographic imaging. Variables analyzed included patient demographics, surgical parameters, intraoperative complications, postoperative complications, and neurosensory recovery outcomes.

Neurosensory evaluation

**Figure 1:**
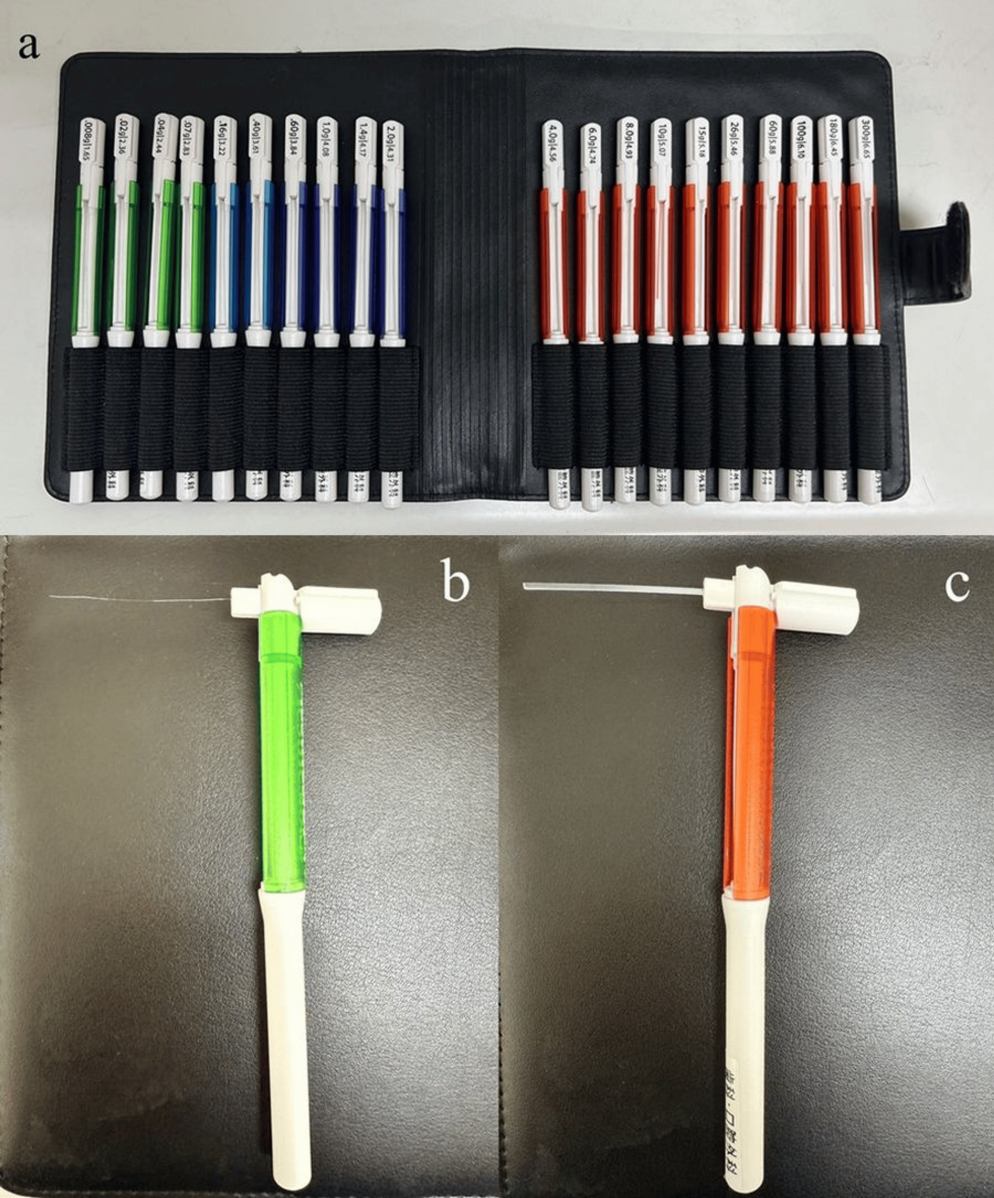
Semmes-Weinstein (SW) monofilament testing instruments for sensory evaluation (a) SW monofilament tester kit containing multiple calibrated nylon filaments for comprehensive sensory evaluation. The complete set includes monofilaments ranging from 1.65 to 6.65, each exerting a specific target force for graded tactile threshold testing. (b) Fine-calibrated 1.65 monofilament designed to detect subtle changes in tactile sensation. This probe applies a force of 0.008 grams and is used as the standard for normal light touch perception in clinical neurological evaluations. (c) The 6.65 monofilament (300 gram-force) used for general sensation screening. Inability to perceive this filament is considered indicative of severe sensory impairment. Device used: Semmes-Weinstein (SW) monofilament device (Sakai Medical Co., Ltd., Tokyo, Japan).

Sensory function was assessed using a standardized Semmes-Weinstein (SW) monofilament testing protocol at one week, one month, two months, three months, and six months postoperatively (Figure 1).

Board-certified oral and maxillofacial surgeons experienced in SW monofilament testing protocols performed neurosensory assessments. Following established clinical guidelines, all examiners applied the monofilaments perpendicular to the skin surface for approximately 1.5 seconds until filament bending occurred.

Each test site was assessed two to three times per examination. Sensation was recorded as normal if the patient detected the SW 1.65 monofilament in the majority of trials at that site. Normal sensation was defined as the detection of the SW 1.65 (0.008 g) monofilament. Severe hypoesthesia was defined as the failure to detect the SW 4.56 (4.0 g) monofilament or higher. Recovery was defined as the time point at which SW 1.65 detection was restored. Testing was performed bilaterally in standardized regions: the infraorbital nerve distribution for Le Fort I osteotomy cases and the IAN distribution for SSRO cases [14-16].

Statistical analysis

Analyses were performed using Easy R or EZR (R version 4.3.1; R Foundation for Statistical Computing, Vienna, Austria, https://www.R-project.org/) [17]. Categorical variables were compared using the chi-square test or Fisher's exact test. Continuous variables were analyzed with Student's t-test or the Mann-Whitney U test, as appropriate. Correlations between ordinal variables were examined using Spearman's rank correlation coefficient. Independent risk factors were identified using multivariate logistic regression. Statistical significance was set at *p*<0.05.

## Results

Patient demographics

Table 1 shows the demographic characteristics and surgical features of the patients.

**Table 1:** Patient demographics and surgical characteristics. SSRO: sagittal split ramus osteotomy; SD: standard deviation. Values are presented as mean ± SD or n (%). Statistical significance was determined using Student's t-test for continuous variables (age) and chi-square test for categorical variables (sex, surgical movement, magnitude of movement).

Characteristic	Le Fort I (n=225)	SSRO (n=354)	Total (n=579)	p value
Age (years), mean ± SD	24.3 ± 6.8	26.1 ± 7.2	25.2 ± 7.0	0.089
Sex, n (%)				
Male	92 (40.9%)	142 (40.1%)	234 (40.4%)	0.894
Female	133 (59.1%)	212 (59.9%)	345 (59.6%)	
Surgical movement, n (%)				
Advancement	112 (49.8%)	96 (27.1%)	208 (35.9%)	<0.001
Setback	113 (50.2%)	258 (72.9%)	371 (64.1%)	
Magnitude of movement, n (%)				
≥5 mm	100 (44.4%)	164 (46.3%)	264 (45.6%)	0.678
<5 mm	125 (55.6%)	190 (53.7%)	315 (54.4%)	

Of the 642 consecutive patients who underwent orthognathic surgery during the study period, 579 cases with a complete six-month follow-up met the selection criteria. Of those, 225 underwent Le Fort I osteotomy and 354 underwent SSRO. The mean age was 24.3 ± 6.8 years for the Le Fort I group and 26.1 ± 7.2 years for the SSRO group. The male-to-female subject ratio was approximately 1:1.5 in both groups.

Intraoperative complications

**Figure 2:**
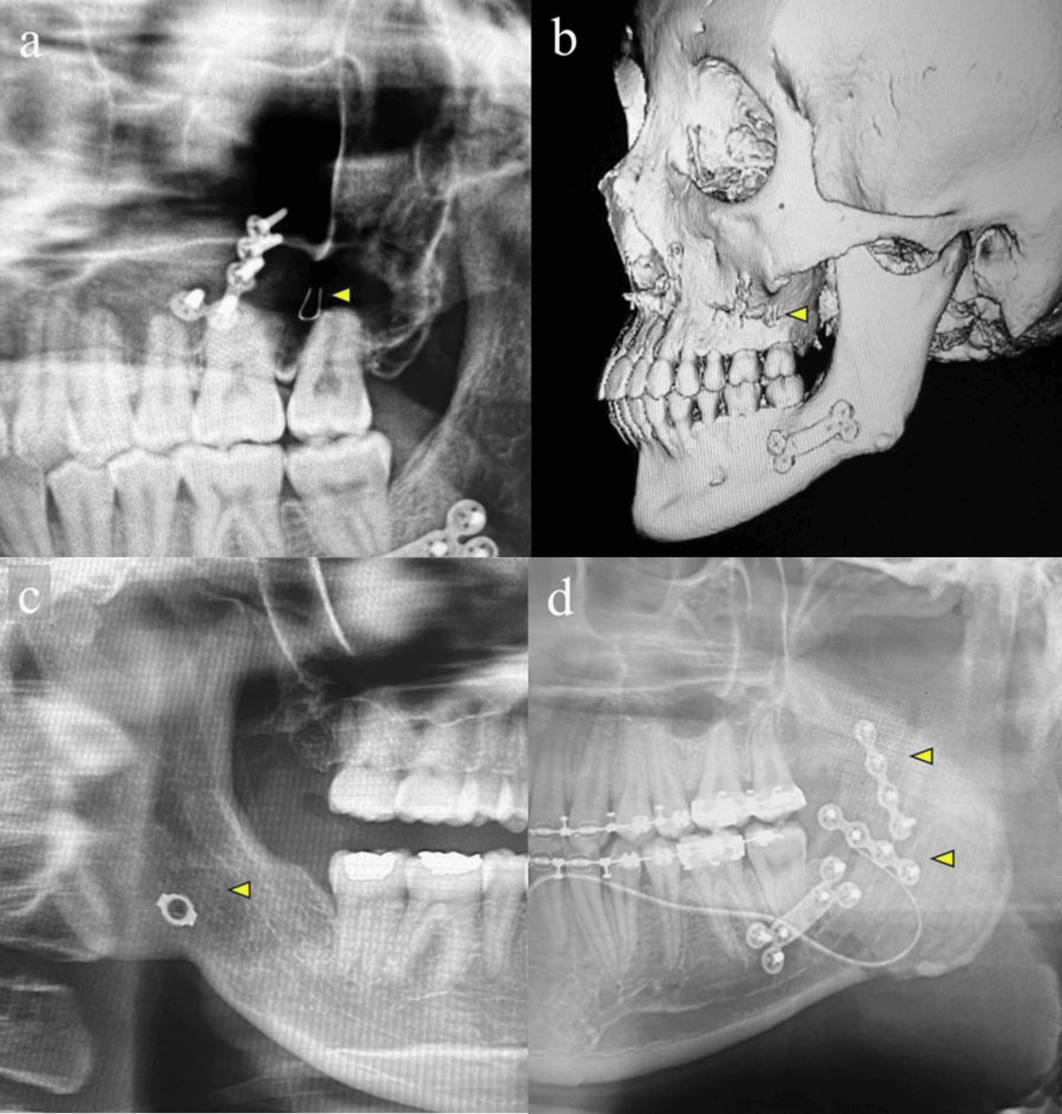
Representative cases of complications following orthognathic surgery SW monofilament testing instruments for sensory evaluation Figures 2a, 2c, 2d are panoramic X-ray images, and Figure 2b is a 3D multi-detector computed tomography (MDCT) reconstruction. Case 1 (a, b): Inadvertent retention of a metal wire for intermaxillary fixation along the buccal bone wall of the left posterior maxilla. (a) A panoramic radiograph showing the retained metal wire (arrowhead). (b) Three-dimensional MDCT reconstruction showing the same retained metal wire (arrowhead). Case 2 (c): Inadvertent intrusion of the cut edge of a metal fixation plate into the lateral space of the mandibular ramus (arrowhead). Case 3 (d): Unfavorable split(s) of the anterior margin of the mandibular ramus (arrowheads).

The overall intraoperative complication rates were low in both groups (Table 2, Figure 2). 

**Table 2:** Comparison of the intraoperative, postoperative, and rare complications after Le Fort I osteotomy and SSRO ENT: ear–nose–throat; PONV: postoperative nausea and vomiting; SSRO: sagittal split ramus osteotomy. Data are presented as number and percentage of affected cases. Neurosensory disturbances include paresthesia and hypoesthesia. Rare complications occurred in <1% of cases.

Procedure group	Complication	Cases/total	Percentage
Intraoperative complications			
Le Fort I	Foreign body migration (orthodontic wire)	1/225	0.4%
SSRO	Unfavorable splits	6/354	1.7%
	Excessive bleeding	1/354	0.3%
	Foreign body migration (orthodontic wire)	1/354	0.3%
	Thermal burn at oral commissure	1/354	0.3%
Postoperative complications			
Le Fort I	Infraorbital nerve disturbance	36/225	16.0%
	Maxillary sinus mucosal thickening	32/225	14.2%
	Nasal obstruction requiring ENT consultation	6/225	2.7%
SSRO	Inferior alveolar nerve disturbance, left side	139/354	39.3%
	Inferior alveolar nerve disturbance, right side	112/354	31.6%
	Metal plate fracture (without displacement)	4/354	1.1%
Rare complications	Postoperative agitation or panic; Extrapyramidal symptoms; Urethral complications; Hyperamylasemia; PONV	1-5	<1%

**Figure 3:**
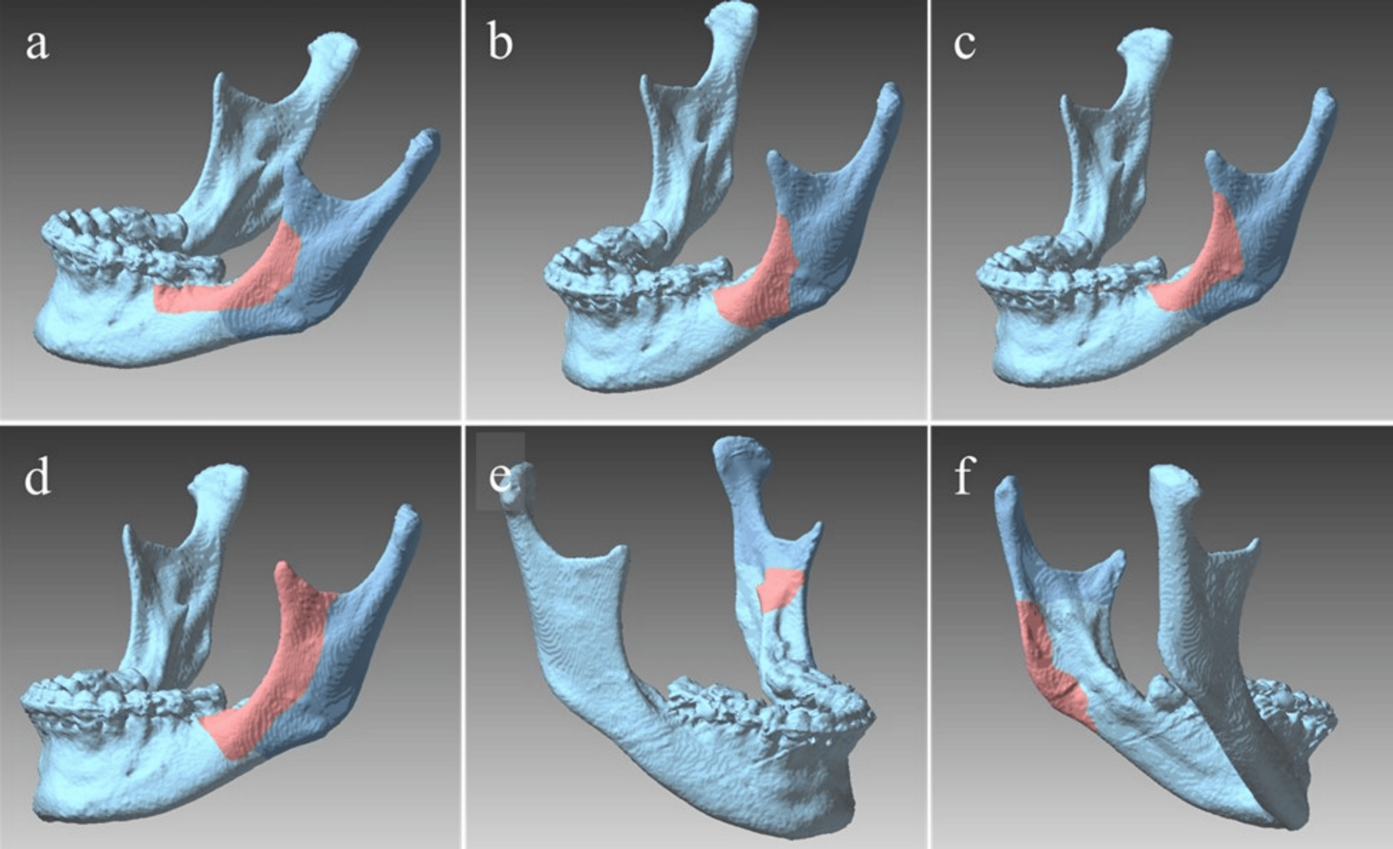
Three-dimensional illustrations of unfavorable split patterns in sagittal split ramus osteotomy (SSRO) Representative six cases of unfavorable split patterns following SSRO. The dark blue areas represent the standard SSRO bone segments, and the red areas indicate the unfavorable split regions. (a) Anterior border of the mandibular ramus; (b) Anterior border with inferior extension; (c) Longitudinal split of the anterior border; (d) Extension to the mandibular notch; (e) Inclusion of the mandibular lingula in the lateral segment; (f) Incomplete split at the mandibular angle. Image credit: Original image from the current study using a POLYGONALmeister V9 (UEL Corp., Tokyo, Japan).

In the Le Fort I group, a single case (0.4%) of foreign body migration involving orthodontic wire was observed. In the SSRO group, unfavorable splits represented the most frequent complication (six cases, 1.7%) (Figure 3), followed by excessive bleeding (one case, 0.3%), foreign body migration (one case, 0.3%), and commissural thermal burn (one case, 0.3%).

Postoperative complications

Postoperative complications were more frequent than intraoperative events, with neurosensory disturbances representing the most common adverse outcome. In the Le Fort I group, infraorbital nerve paresthesia occurred in 36 patients (16.0%), maxillary sinus mucosal thickening at six months in 32 (14.2%), and severe nasal obstruction requiring ear, nose, and throat consultation in six (2.7%). In the SSRO group, IAN disturbances were more prevalent on the left side (139 cases, or 39.3%) than on the right (112 cases, or 31.6%), and frequently involved both sides. Four patients (1.1%) had a metal plate fracture without displacement.

Neurosensory recovery

Postoperative neurosensory recovery rates were high in both procedural groups (Table 3).

**Table 3:** Neurosensory recovery outcomes six months after the procedure IAN: inferior alveolar nerve; SSRO: sagittal split ramus osteotomy. Data are presented as n (%). Recovery rate represents the proportion of patients with neurosensory disturbance who achieved normal sensation within six months postoperatively. Majority of recoveries (>85%) occurred within 3 months of surgery.

Procedure group	Total affected cases (n)	Recovery at 6 months (n)	Recovery Rate (%)	Primary recovery window
Le Fort I (Infraorbital nerve)	36	34	94.4%	>85% within 3 months
SSRO - Left (IAN)	139	129	92.8%	>85% within 3 months
SSRO - Right (IAN)	112	104	92.9%	>85% within 3 months
SSRO combined	251	233	92.9%	>85% within 3 months

In the Le Fort I osteotomy group, 34 out of 36 patients (94.4%) with infraorbital nerve disturbance achieved sensory recovery within six months. The SSRO group demonstrated comparable recovery rates, with 129 out of 139 (92.8%) affected left sides and 104 out of 112 (92.9%) affected right sides showing restoration of normal sensation. Overall, 233 of 251 affected sides (92.9%) in the SSRO group recovered normal sensation within the follow-up period. Notably, the majority (>85%) of patients who regained normal sensation did so within the first three months postoperatively, demonstrating a favorable and predictable recovery trajectory in both groups.

Risk factor analysis

Statistical analysis identified key risk factors for postoperative neurosensory complications. Table 4 summarizes infraorbital nerve paresthesia in the Le Fort I osteotomy group (n=225).

**Table 4:** Incidence of infraorbital nerve disturbance following Le Fort I osteotomy (n=225) Data are presented as n (%). Occlusal plane adjustments include non-sagittal movements such as rotation, asymmetry correction, and changes to the occlusal plane. Statistical significance was determined using chi-square test or Fisher's exact test. All p values >0.05. No significant associations found between patient demographics and the occurrence of infraorbital nerve disturbance.

Variable	Category	Disturbance (+)	Disturbance (−)
Total		36 (16.0%)	189 (84.0%)
Sex	Male	7 (11.9%)	52 (88.1%)
	Female	29 (17.5%)	137 (82.5%)
Age (years)	<25	19 (15.8%)	101 (84.2%)
	≥25	17 (16.2%)	88 (83.8%)
Type of surgical movement	Setback	10 (15.4%)	55 (84.6%)
	Advancement	13 (19.4%)	54 (80.6%)
	Occlusal plane adjustment etc.	13 (14.0%)	80 (86.0%)

Table 5 summarizes inferior alveolar nerve (IAN) disturbance by side in the SSRO group (n=354 per side).

**Table 5:** Incidence of inferior alveolar nerve disturbance after sagittal split ramus osteotomy (SSRO; n=354 per side) Data are presented as n (%). Statistical significance was determined using chi-square test or Fisher's exact test (*p*<0.05). Occlusal plane rotation cases without specific advancement/setback classification were included in the type analysis.

Variable	Category	Left side (n=354)			Right side (n=354)		
		Disturbance (+)	Disturbance (−)	*p* value	Disturbance (+)	Disturbance (−)	*p* value
Total		139 (39.3%)	215 (60.7%)		112 (31.6%)	242 (68.4%)	
Sex	Male	75 (52.8%)	67 (47.2%)	0.933	33 (30.5%)	75 (69.5%)	0.899
	Female	64 (30.2%)	148 (69.8%)		79 (32.1%)	167 (67.9%)	
Age	<25 years	74 (39.1%)	115 (60.9%)	0.974	57 (30.1%)	132 (69.9%)	0.921
	≥25 years	65 (39.4%)	100 (60.6%)		55 (33.3%)	110 (66.7%)	
Type of surgical movement	Setback	106 (38.1%)	172 (61.9%)	0.347	33 (30.6%)	75 (69.4%)	0.473
	Advancement	33 (43.4%)	43 (56.6%)		79 (32.1%)	167 (67.9%)	
	Occlusal plane rotation etc.	0 (0%)	0 (0%)		0 (0%)	0 (0%)	

In the Le Fort I group, no significant associations were found between patient demographics and the occurrence of infraorbital nerve paresthesia. In the SSRO group, mandibular advancement of ≥5 mm emerged as the strongest risk factor for bilateral IAN disturbance (left side: *p*=0.000292; right side: *p*=0.00196). No significant correlations were observed with patient age, sex, or setback procedures (all *p*>0.05).

Rare complications

**Figure 4:**
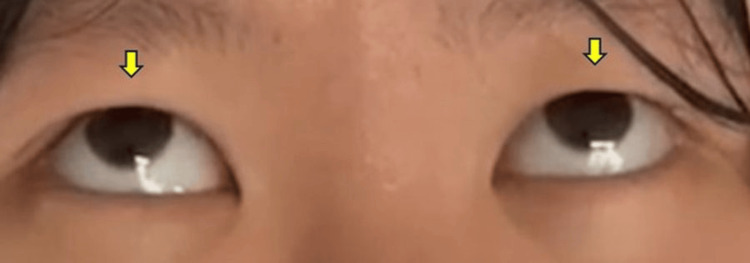
Drug-induced extrapyramidal complication Clinical photograph showing sustained upward deviation of both eyes (oculogyric crisis, arrow) following intravenous patient-controlled analgesia administration.

Several uncommon complications were observed, each occurring in only one or a few cases. These included postoperative agitation, extrapyramidal symptoms with oculogyric crisis associated with intravenous patient-controlled analgesia administration (Figure 4), urethral complications, hyperamylasemia, and postoperative nausea and vomiting.

## Discussion

The complication patterns observed in this study are broadly consistent with contemporary reports. The incidence of IAN disturbances in the SSRO group (39.3% on the left side and 31.6% on the right side) was higher than the 20%-30% rates reported in multi-institutional studies [3,15,18]. This discrepancy may be explained by the hospital’s role as a tertiary referral center, where patients often present with complex anatomical variations and comorbidities. Technical considerations may also contribute: the use of the Obwegeser-Dal Pont technique and the application of augmented titanium plate fixation with additional screws to enhance rigidity may inadvertently increase mechanical compression in the region of the IAN. Comparative risk assessments in orthognathic cohorts emphasize the importance of standardized perioperative evaluation protocols to systematically quantify the risk of nerve injury and enable evidence-based patient counseling [18].

Neurosensory recovery outcomes in this cohort were favorable. At six months, recovery rates reached 94.4% for infraorbital nerve disturbances following Le Fort I osteotomy and 92.9% for IAN disturbances following SSRO. These rates compare favorably with historical data, underscoring the impact of recent surgical innovations. The introduction of ultrasonic osteotomy devices and refined periosteal elevation techniques appears to have minimized trauma to neurovascular structures, thereby enhancing functional recovery [19,20].

The statistically significant association between mandibular advancement of ≥5 mm and an increased risk of IAN disturbance is consistent with previous studies that identified advancement procedures as major risk factors [21,22]. The likely mechanism involves increased tension on the neurovascular bundle at the mandibular foramen and potential compression between bone segments during rigid fixation.

Several potential confounding factors must be considered when interpreting these findings. First, although all surgeries were performed by experienced maxillofacial surgeons, variations in surgeon proficiency and operative technique may contribute to heterogeneous outcomes. While our multivariate analysis identified mandibular advancement as the strongest independent risk factor, unmeasured operator-level variables, such as dissection technique, nerve identification protocols, and intraoperative decision-making, may also influence complication rates. Second, although all cases employed titanium miniplate fixation, variations in the number of screws used, plate positioning, and compression force were not documented and may affect the risk of compression-related injury. Third, excluding 63 patients (9.8%) introduces potential selection bias. If patients who experienced complications were more likely to discontinue follow-up, our incidence estimates would underestimate the true burden of complications. Conversely, if patients who recovered early were less likely to adhere to follow-up, our estimates would be inflated. The direction and magnitude of this bias cannot be determined from retrospective data. Finally, the six-month follow-up period may not capture late neurosensory recoveries, which could lead to an underestimation of ultimate recovery rates.

Unfavorable splits were observed in six cases, all occurring on the left side. This laterality may reflect the influence of surgeon ergonomics. For right-handed surgeons, the left surgical field often presents a less favorable working angle and restricted visibility compared with the right, which can reduce precision during osteotomy and fixation. The operating surgeon stands on the patient's right side during osteotomy procedures, regardless of whether the osteotomy is performed on the left or right side. These ergonomic challenges may explain both the higher incidence of IAN paresthesia on the left side (39.3% vs. 31.6% on the right) and the observed laterality of unfavorable splits [23,24]. The incidence of unfavorable splits in this series (1.7%) is comparable to or lower than rates reported in previous literature [25,26]. Preventive strategies remain critical. Ensuring complete osteotomy prior to split (fracture) initiation reduces the risk of poor splits, while preoperative computed tomography evaluation of mandibular ramus morphology and third molar status may help identify patients at increased risk [27,28].

Other complications included maxillary sinus mucosal thickening (14.2%) and metal plate fracture (1.1%). For maxillary sinus mucosal thickening, our previous investigations have established management protocols [29], and no patients exhibited postoperative worsening. The occurrence of extrapyramidal symptoms in this series represents a rare but recognized adverse effect of antiemetic agents used for postoperative nausea and vomiting prophylaxis. This highlights the importance of comprehensive perioperative management in major orthognathic procedures [30-32].

This study has several limitations. Its retrospective design and single-center scope may restrict generalizability, while the relatively high complication rates may reflect the referral center’s complex case mix rather than suboptimal surgical outcomes. Future prospective, multicenter studies employing standardized evaluation protocols are warranted to provide more robust evidence. Emerging technologies - including computer-assisted surgical planning with three-dimensional modeling [33-36] and intraoperative navigation integrating advanced imaging with virtual planning - hold promise for further enhancing surgical precision and reducing complication rates. Inter-rater reliability among multiple examiners was not formally evaluated over the five-year study period. Although SW monofilament testing has been shown to be reliable in previous studies, potential variability in measurement among examiners in our clinical setting cannot be ruled out. This is an inherent limitation of the retrospective design that should be addressed through rigorous standardization and inter-rater reliability testing in future prospective investigations. It was not feasible to blind the examiner in this retrospective study. Sensory assessments were conducted by the treating clinicians during standard postoperative follow-up, which precluded a blinded evaluation. Although this may introduce assessment bias, it is neither feasible nor ethical to blind treating physicians to surgical details in routine clinical practice. Future prospective studies employing independent, blinded assessors would strengthen the study's methodology.

## Conclusions

This retrospective study shows that although orthognathic surgery is associated with a low risk of intraoperative complications, it has a notable incidence of transient neurosensory deficits. Majority of patients achieved sensory recovery within six months. These outcomes compare favorably with historical reports and are consistent with current surgical protocols. We identified a statistically significant association between mandibular advancement of at least 5 mm and an increased risk of IAN disturbance. However, the retrospective design and potential residual confounding by unmeasured variables (ramus anatomy, specific fixation techniques, and operator experience) preclude definitive causal inference. These findings may reflect the case mix of our tertiary referral center and may not be fully generalizable to community practice settings. Prospective studies are needed to confirm these associations. Rigorous postoperative sensory monitoring combined with selective intraoperative nerve navigation in high-risk cases may further reduce the risk of permanent nerve injury.
